# Intention-Action Conflict EEG-Hand Kinematics Dataset for Unimanual Control under Congruent and Incongruent Conditions

**DOI:** 10.1038/s41597-026-07146-x

**Published:** 2026-04-22

**Authors:** Mengpu Cai, Rongrong Fu, Yaodong Wang, Bin Lu, Saiwei Guo, Fangyao Xu

**Affiliations:** 1 School of Electrical Engineering, Yanshan University, Qinhuangdao, 066004 China; 2 Hebei Key Laboratory of Measurement Technology and Instrumentation, Yanshan University, Qinhuangdao, 066000 Hebei Province China; 3 Key Laboratory of Intelligent Control and Neural Information Processing, Ministry of Education, Yanshan University, Qinhuangdao, 066004 Hebei Province China; 4 School of Computer Science, Wuhan University, Wuhan, 430072 China

## Abstract

The Intention-Action Conflict EEG-Hand Kinematics Dataset (IACKD) is a joint resource for studying congruent and incongruent intention-action conditions during unimanual control. It comprises 7,040 trials from 15 participants. Each trial includes a 1-s pre-movement period and a movement-execution period. A target frame and a controllable ball define the task; ball color cues congruency (red = same direction; yellow = opposite). EEG was recorded with a 32-channel Compumedics Neuroscan system at 1024 Hz, and 3-D hand trajectories were captured with Leap Motion at 170 Hz. Streams are time-aligned, and complete preprocessing and alignment scripts are provided. Technical validation includes readiness potentials at C3/C4/Cz, μ/β-band ERD/ERS with post-movement β rebound, sub-30-ms cross-modal residuals on >99.375% of trials, and expected completion-time differences between congruent and incongruent conditions. IACKD is intended for reuse in intention decoding, continuous trajectory decoding, and evaluation of decoder robustness under conflict or perturbed feedback.

## Background & Summary

Decoding upper-limb kinematics from scalp electroencephalography (EEG) is neurophysiologically well-grounded^1,2^. Goal-directed actions reliably elicit central motor signatures such as readiness potentials (RPs)^3^ and μ/β-band (8–30 Hz) event-related desynchronization/synchronization (ERD/ERS)^4^, which have been leveraged across both motor-imagery (MI) and motor-execution (ME) paradigms for non-invasive brain-computer interfaces (BCIs) and trajectory decoding^5–7^.

Broadly, the literature spans (i) MI-based intention decoding—highly relevant to post-stroke rehabilitation^8^ and assisted control^9^—and (ii) ME-based decoding of continuous trajectories or end-effector states during real movements^10–12^.

Nevertheless, most datasets implicitly assume intention-action congruency, whereas visuomotor/intention-action conflicts are common in real interaction—for example when teleoperation introduces communication delays^13^, or when VR/AR induces motion-to-photon latency and spatial mis-registration^14^—thereby altering sensorimotor integration and cortical dynamics. Neuroimaging and EEG studies show that such conflicts engage fronto-parietal control networks and modulate motor rhythms, implying more stringent requirements for robust EEG-based control^15^.

We present an IACKD dataset that explicitly contrasts congruent versus incongruent intention-action conditions during unimanual control. It comprises (i) pre-movement EEG and (ii) EEG plus 3-D hand kinematics during movement execution. The paradigm uses a target frame and a controllable ball. The ball color cues congruency (red = hand and ball move in the same direction; yellow = opposite). In this context, “intention” refers to the abstract task goal of reaching the target frame, whereas “congruent” and “incongruent” describe whether the required hand movement direction matches or opposes the visually cued ball motion. Unlike paradigms that introduce unexpected perturbations to elicit prediction error-driven adaptation, the present design focuses on motor control under predefined and explicitly instructed mappings, thereby inducing rule-based, anticipated visuomotor conflicts that allow systematic investigation of motor preparation and execution under known remapping conditions. The dataset includes 15 participants, totaling 7,040 trials (6,671 valid). Each trial provides a 1-s pre-movement interval, followed by continuous recording of EEG and hand-center 3-D trajectories during execution.Hand motion was captured with a Leap Motion sensor^16^, whose tracking precision has been quantitatively characterized in rehabilitation-style tasks^17^.

This resource is valuable on three fronts. Paradigm: it supplies a rare, well-controlled intention-action conflict EEG-kinematic dataset. Methodology: it spans RP and μ/β time-frequency dynamics, enabling cross-stage modeling from preparation to execution. Application: robustness to altered visuomotor mappings is critical for assistive control systems. Rather than serving as a direct benchmark for everyday prosthetic manipulation, IACKD provides a controlled model of simple pointing-like movements under known congruent and incongruent visuomotor mappings, which can be used to study how EEG-based decoders cope with systematic remapping and rule-based conflicts.

## Methods

### Participants

Fifteen healthy volunteers (11 males and 4 females, aged 23–27 years, mean age ≈ 24.3 years), all native Chinese university students, participated in the experiment. Handedness was assessed using the Edinburgh Handedness Inventory, and all participants were right-handed. All participants reported normal or corrected-to-normal vision and normal color vision, which was essential given the color-based cues used in the experimental paradigm. None of the participants had a history of neurological, psychiatric, or musculoskeletal disorders, and none were taking psychoactive or neuroactive medication at the time of the experiment. Before the experiment, participants were fully informed of the study procedure and received training to familiarize themselves with the experimental tasks. To ensure anonymity, each participant was labeled as s01-s15. All experimental procedures complied with the ethical standards of the Declaration of Helsinki and were approved by the Ethics Committee of the First Hospital of Qinhuangdao (Approval No. 2024G006). The committee approved both the experimental protocol and the anonymized data sharing and public release of the dataset for scientific research purposes. Written informed consent was obtained from all participants prior to data collection. The consent form explicitly included permission for anonymized data storage, sharing, and open public release of the dataset for scientific research purposes.

This dataset primarily reflects healthy young adult participants of Chinese ethnicity, with a predominance of male subjects. Therefore, caution should be exercised when extrapolating the findings to other age groups, cultural backgrounds, or clinical populations. Future extensions of the dataset aim to include a broader demographic range to enhance generalizability.

### Experimental procedure

**Fig. 1:**
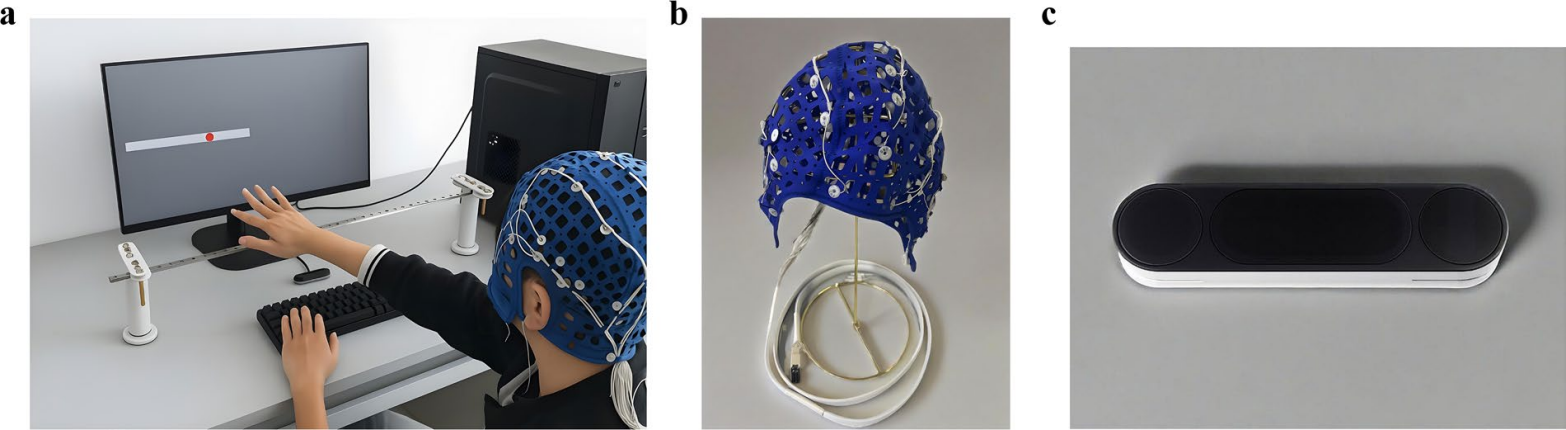
Experimental setup and acquisition devices. (**a**) Experimental setup showing the participant performing the task while wearing a 32-channel EEG cap; the Leap Motion sensor was placed in front of the keyboard, and a guiding rail was positioned behind it to constrain hand movement. (**b**) The EEG cap with a 32-channel electrode configuration. (**c**) The Leap Motion Controller for 3-D hand motion tracking. *The participant shown in this figure provided written informed consent for the open publication of the image*.

**Fig. 2:**
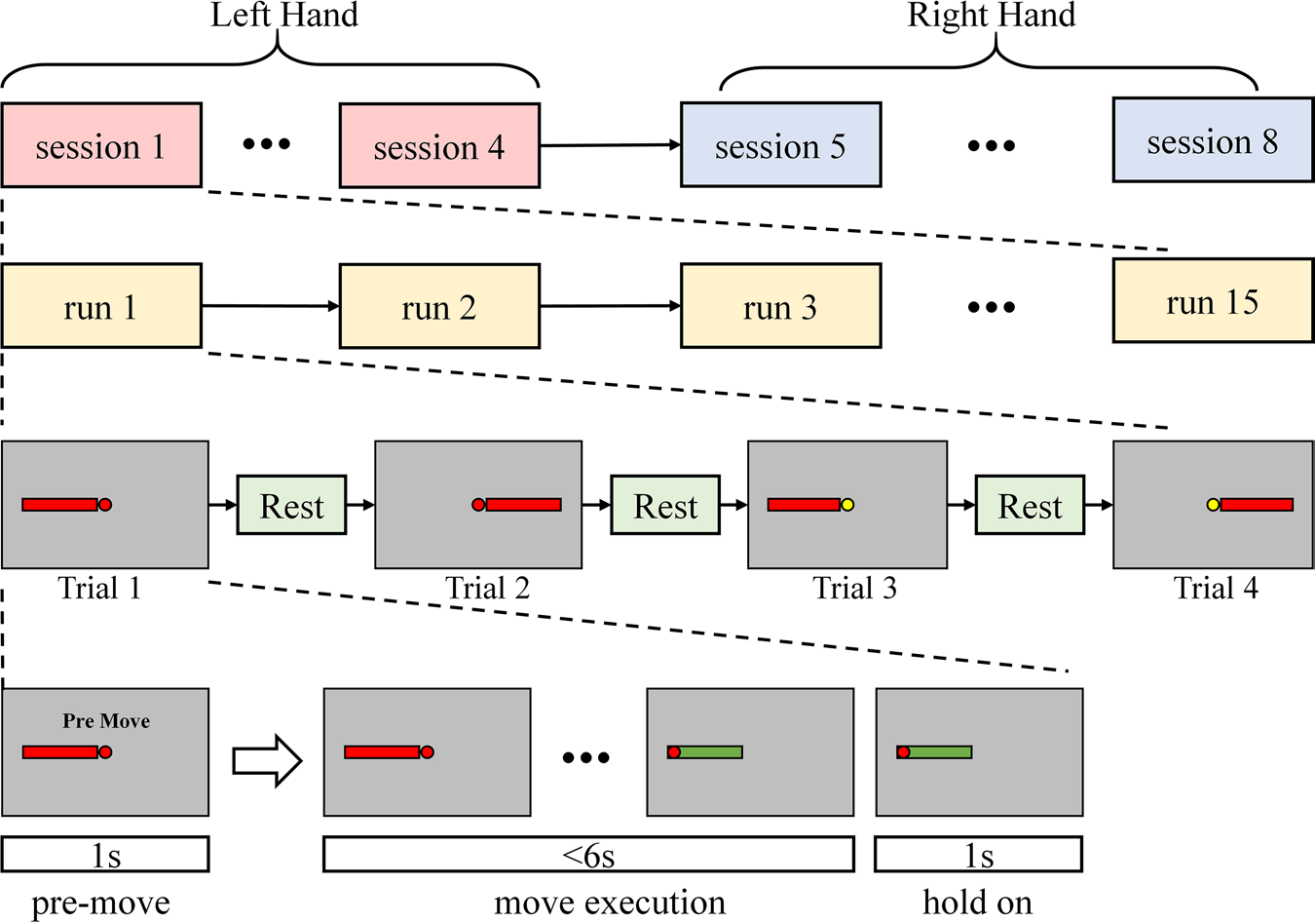
Experimental workflow and trial structure. Schematic of the overall procedure and the two task stages: *pre-movement* (−1 to 0 s) and *movement execution* (0 s to target boundary), including the congruent/incongruent cueing logic.

An overview of the experimental setup and acquisition devices is provided in Fig. 1, and the detailed experimental procedure and trial timeline are illustrated in Fig. 2. After indicating readiness, an instruction screen appeared to familiarize participants with the overall procedure. Two equivalent experimental configurations were adopted in this study. In the first configuration, each participant completed eight sessions (four with the left hand and four with the right hand), with 15 runs per session. In the second configuration, each participant completed twelve sessions (six with the left hand and six with the right hand), with 10 runs per session. Both configurations resulted in the same total number of runs and trials per participant. Participant s01 was the first subject recruited and was tested during the pilot phase of the experiment. For this participant, eight sessions were conducted, each consisting of 10 runs. This preliminary setting was used to evaluate task feasibility and physical workload. Based on the feedback that 10 runs per session were relatively light, the two standardized configurations described above were subsequently established. Participants s04 and s05 chose the twelve-session configuration to reduce physical fatigue, as shorter sessions were perceived as less physically demanding. The remaining participants completed the eight-session configuration. Each session consisted of 15 runs (10 runs for participants 1, 4, and 5), and each run included four trials, resulting in 60 trials per session. After completing each trial, the participant initiates the next trial by pressing a key.

At the beginning of each trial, the screen displayed a red rectangular target frame and a red or yellow ball, forming four possible combinations. The rectangular frame appeared either to the left or right of the screen center, indicating the target movement direction for that trial. The ball’s position was controlled in real time using a Leap Motion device. The ball always started at the screen center. When the ball was red, the hand movement direction was consistent with the ball’s movement direction; when the ball was yellow, the ball moved in the opposite direction to the participant’s hand movement.

Each trial comprised two stages: a pre-movement stage and a movement-execution stage. During the pre-movement stage, the words *“Pre Move”* appeared above the ball for 1 s, while participants could not control the ball’s motion. This phase was designed to examine EEG activity related to motor intention before the actual movement onset. When the *“Pre Move”* cue disappeared, participants controlled the ball via hand movement, guiding it from the center to the inner boundary of the target frame. Once the ball touched the boundary, the frame changed color from red to green. Participants were instructed to keep the ball in contact with the boundary for about 1 s, after which the trial was automatically terminated by the system. Trials were considered invalid if the target boundary was not reached within 6 s.

The entire experimental paradigm was implemented using the PsychoPy^18^ framework in Python. To facilitate reproducibility and reuse, the complete PsychoPy experiment scripts have been made publicly available at: https://github.com/Boketto1/IACKD.

In total, each participant performed 480 trials, resulting in 7,200 trials across all participants, of which 7,040 were recorded and 6,671 were retained as valid after quality control. The average duration of one experimental session (15 runs) was approximately 440.930 seconds, and the complete experimental procedure for a single participant lasted about 3527.458 seconds on average.

### Data acquisition

Participants were seated comfortably in front of a 24-inch monitor (resolution: 1920 × 1080 pixels, refresh rate: 60 Hz) at an approximate viewing distance of 60–70 cm. Under this setup, the virtual ball (50 × 50 pixels), initially presented at the screen center, subtended a visual angle of approximately 1.1°–1.3°. The rectangular target frame (900 × 50 pixels) subtended approximately 20.1°–23.4° in width and 1.1°–1.3° in height, depending on the viewing distance. In each trial, the ball moved horizontally from the center toward the left or right target boundary over a displacement of 900 pixels, corresponding to approximately 20.1°–23.4° of visual angle. During each task, only one hand was used to perform the movement, while the other hand was placed naturally on the table to minimize large-scale upper limb motion. For consistency of motor execution, participants were instructed to keep the palm open and relaxed, with the wrist maintained in a neutral position without flexion. Hand motion was mainly generated by elbow movement, while wrist and finger movements were minimized. The task therefore approximated a constrained two-dimensional pointing movement in the horizontal plane with limited degrees of freedom. This posture and movement pattern were standardized across participants during the training session before formal data collection. To avoid fatigue, participants were allowed to take self-paced breaks after each trial and longer rest periods between sessions. All participants reported no significant discomfort or fatigue during the experiment.

EEG data were recorded using a 32-channel EEG cap positioned according to the international 10–20 system, connected to a Compumedics Neuroscan amplifier system. The cap was positioned with the Cz electrode at the center of the scalp. Before each run, EEG-compatible conductive gel was applied and electrode impedance was measured for all electrodes, confirming that all impedances were below 10 kΩ at the start of the run. Impedance was checked again after each run to monitor recording quality. EEG signals were continuously acquired at a sampling rate of 1024 Hz.

For each trial, the experimental system recorded task-related information at the screen refresh rate (60 Hz), including the global experimental time, trial index, 2D hand position (x and y coordinates) used for online ball control, ball position and color, target movement direction, and whether the ball contacted the target boundary.

During the movement execution stage, Leap Motion was employed as an independent high-resolution kinematic recording system to continuously capture three-dimensional hand trajectories (x, y, z) at a sampling rate of 200 Hz, ensuring precise temporal alignment between motion and EEG recordings. Specifically, only the palm center position provided by the Leap Motion skeletal tracking model was used as the kinematic representation of the hand. This point corresponds to the geometric center of the palm and is commonly adopted in kinematic studies^2,12^ as a stable proxy for overall hand position. No finger- or joint-level kinematics were included in the released dataset. The kinematic data were expressed in the device-centered coordinate system of the Leap Motion sensor, following its native convention (x-axis to the right, y-axis upward, and the z-axis perpendicular to the sensor plane).

### Data Pre-processing

EEG data were preprocessed in MATLAB R2020b using EEGLAB v2025.0.0^19^ and ERPLAB 12.01 to ensure data quality and consistency across participants. Two datasets were processed separately, corresponding to the pre-movement and movement execution stages, because these two stages reflect distinct neurophysiological processes and are characterized by different spectral and artifact properties. Although a unified preprocessing pipeline applied to the continuous EEG is possible in principle, we adopted stage-specific preprocessing strategies to better accommodate the different frequency characteristics and contamination sources of the pre-movement and movement execution phases.

For the pre-movement data, where participants did not perform overt movements, muscle and ocular artifacts were first removed to retain only neural activity related to movement intention. This stage may involve a relatively broad frequency range, including low-frequency readiness potentials and higher-frequency sensorimotor rhythms. Therefore, the EEG recordings were downsampled from 1024 Hz to 200 Hz and bandpass-filtered between 0.1 and 45 Hz using a zero-phase FIR filter. Non-EEG channels (M1, M2, HEOG, VEOG) were removed, and independent component analysis^20^ (ICA, runica, extended mode) combined with ICLabel was applied to identify and reject components classified as ocular or muscular artifacts with a probability threshold of ≥0.90. After ICA-based artifact removal, trials exceeding an amplitude threshold of ±300 μV were rejected to further eliminate residual artifacts. The signals were re-referenced to the common average reference, and epochs were extracted based on event pairs (55-14), with a baseline period from −200 to 0 ms relative to the pre-movement cue onset. This pipeline was implemented in the script preprocess_premotor_55_14.m.

For the movement execution data, which contained actual motor activity and are mainly characterized by μ/β-band (8–30 Hz) event-related desynchronization/synchronization (ERD/ERS), the recordings were downsampled to 100 Hz and filtered between 0.1 and 30 Hz. This narrower band emphasizes physiologically relevant motor rhythms while suppressing high-frequency muscle contamination. ICA was again applied to remove non-neural components, and the data were re-referenced to the average reference. EEG epochs were segmented by event pairs (14 → 1000001) with enforced −200-0 ms baselines, followed by baseline correction and artifact rejection using a ±300 μV amplitude threshold. This process was conducted with the script preprocess_eeg_pipeline.m.

The hand kinematic and ball trajectory data were processed using process_leap_dual_csv.m. Hand 3D trajectories (x, y, z) and ball parameters—including position, color, direction, and hit status—were merged on a per-trial basis and resampled to 100 Hz. To obtain a uniform time base, the kinematic data were first interpolated to a sampling rate of 170 Hz and then resampled to 100 Hz. This sampling rate was chosen as a compromise between temporal resolution and data size, and it is sufficient to capture the dominant frequency content of human hand movements, which typically lies below 10 Hz. Moreover, resampling both EEG (movement execution data) and kinematic signals to 100 Hz facilitates efficient multimodal synchronization and joint analysis. The hand trajectories were smoothed with a 4th-order zero-phase Butterworth filter (4 Hz)^21^, and categorical variables were mapped via nearest-neighbor interpolation. The *hit* event was used to determine the functional endpoint of each trial.

EEG-Leap Motion temporal alignment was performed using align_eeg_leap.m, which matched EEG and Leap trials under order constraints through dynamic programming or tolerance-based greedy pairing. Event anchors (14 ↔ start) and (66 ↔ hit) were used to fit a linear mapping between the two time domains,

$${t}_{{LM}}=a+b{t}_{{EEG}},$$

where *b* was initialized from the median duration ratio and refined through iterative least-squares optimization with outlier rejection. The outliers were defined as trial pairs whose temporal alignment residuals exceeded three standard deviations from the median and were excluded iteratively.

The final calibration and data export were completed using calibrate_pairs_with_events.m and export_eeg_leap_aligned.m. EEG timestamps were projected onto the Leap Motion global time axis using the fitted parameters *(a, b)*, and trajectories were interpolated without extrapolation (out-of-range samples were assigned *NaN*). Interpolation was applied only to the kinematic data, whereas EEG signals were segmented using event markers and were not interpolated. Valid trials were selected based on residual alignment errors and the temporal coverage ratio *cov*, defined as

$$\mathrm{cov}=\frac{{t}_{{LM}}\in [{t}_{\min },{t}_{\max }]}{{t}_{{LM}}}$$

where $[{t}_{\min },{t}_{\max }]$ denotes the valid global-time range of the corresponding Leap Motion trial. Trial selection was performed jointly across modalities. EEG trials failing quality control were discarded together with their corresponding kinematic data, and kinematic trials failing quality control were likewise removed from the EEG dataset. Only EEG-kinematic trial pairs that passed quality checks in both modalities and were successfully aligned were retained in the final dataset. On average, 6.0279 ± 5.1297% of trials were rejected across participants after EEG and kinematic quality control.

Category labels were derived by majority voting within the overlapping time range, and the aligned multimodal dataset was exported as a unified structure for subsequent spectral and decoding analyses.

### Ethics statement

All experimental procedures complied with the ethical standards of the Declaration of Helsinki and were approved by the Ethics Committee of the First Hospital of Qinhuangdao (Approval No. 2024G006). The committee approved both the experimental protocol and the anonymized public sharing of the dataset. Written informed consent was obtained from all participants prior to data collection, and the consent form explicitly included permission for anonymized data storage, sharing, and open publication. All data were de-identified before release to protect participant privacy.

## Data Records

The dataset is publicly available on OpenNeuro^22,23^ under accession number **ds006840** (https://openneuro.org/datasets/ds006840^24^). The repository follows a BIDS-compliant organization for raw EEG data and includes additional source behavioral recordings and derived datasets for EEG-kinematic alignment.

**Fig. 3:**
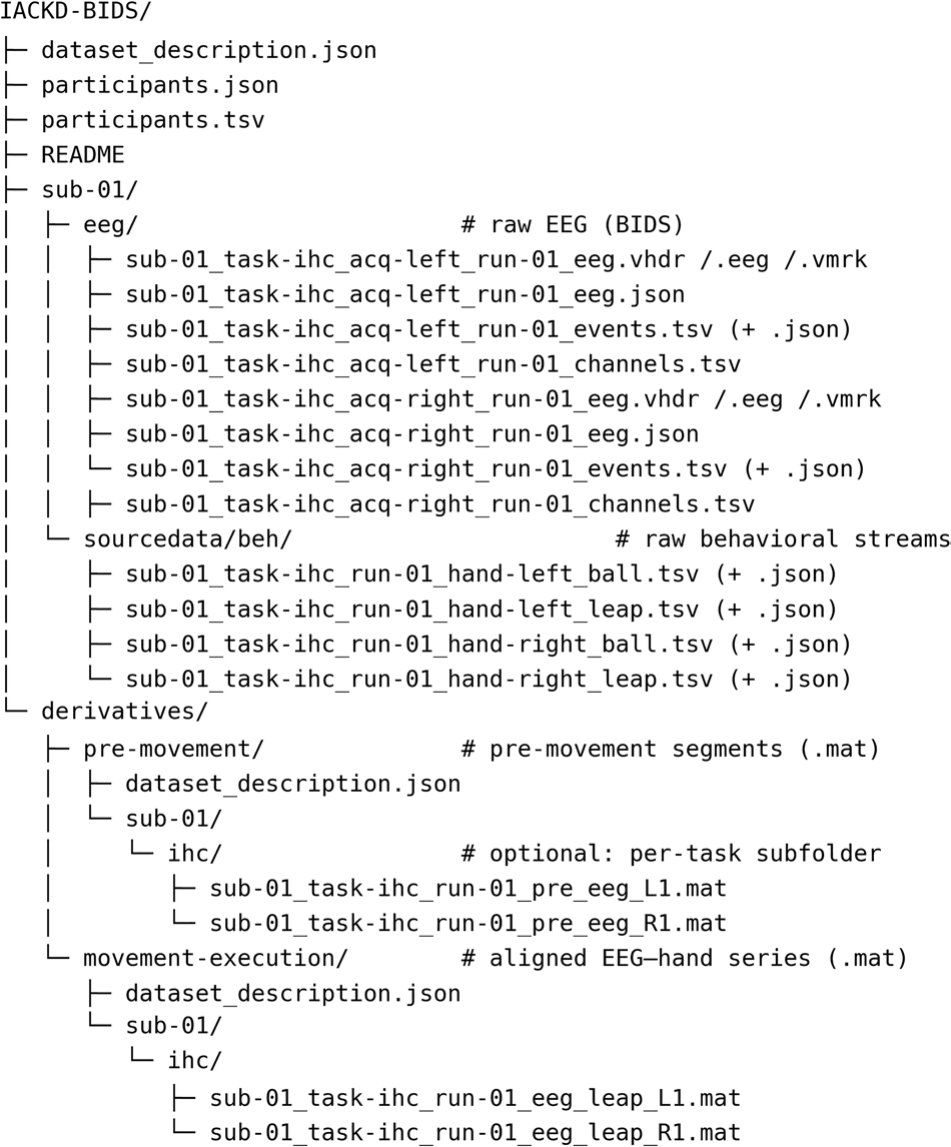
Folder tree of the distributed data. Tree diagram illustrating the BIDS-compatible raw EEG (*/eeg*) with per-run BrainVision files and events.tsv, and the accompanying source behavioral streams (*/sourcedata/beh*) containing screen stimulus (*“ball”*) and hand kinematics (*“leap”*) time series. Filenames and field names are harmonized across subjects and runs to facilitate programmatic reuse.

Data are organized by participant under Datasets/s1-s15 (Fig. 3). Each subject directory includes raw records acquired during the experiment and derived records produced by a standardized processing workflow. Filenames, and field names are harmonized across subjects to facilitate programmatic access.

Table 1 summarizes the content, format, and units of each major component in the dataset, including raw EEG, Leap Motion kinematic data, derived aligned EEG-hand motion files, and pre-movement EEG segments. The inclusion of standardized field names and harmonized file structures enables programmatic access.

**Table 1:** Summary of data files and folders included in the dataset.

File/Folder	Description	Format and Units
IACKD-BIDS/sub-XX/eeg/	BIDS BrainVision EEG per-run with *_events.tsv.	.vhdr/.eeg/.vmrk,.json,.tsv.
IACKD-BIDS/sub-XX/sourcedata/beh/	Source behavioral time series (“ball” screen trajectory; “leap” hand kinematics).	*_ball.tsv.gz, *_leap.tsv.gz ( + .json); mm/px; ignored by validator via.bidsignore.
IACKD-BIDS/derivatives/movement_execution/	Aligned EEG-hand time series per run (struct OUT: EEG, x/y/z_mm, ball_x_pix, timing, etc.).	.mat;EEG µV, 100 Hz; positions mm; pixels px.
IACKD-BIDS/derivatives/pre_movement/	Pre-movement EEG segments (segs_1s/var + metadata).	.mat; EEG µV, 200 Hz; time s.
README (root)	Dataset guide, BIDS notes, quick-start; link to GitHub.	Markdown (UTF-8).

### Raw data

#### EEG

All runs are distributed in BIDS‐compatible BrainVision format under sub-XX/eeg/ (files *_eeg.vhdr/.eeg/.vmrk with sidecar *_eeg.json). Trial timing is provided in *_events.tsv; key markers originate from the CURRY annotations (trial onset corresponds to the 14 event preceded by 55; boundary hit maps to 66, exported as 1000001 in some sessions). Runs are indexed as run-01…, and the moving hand is encoded via the acquisition entity (acq-left/acq-right). For completeness, the source CURRY files (.cdt/.ceo/.dpa) are also retained under sub-XX/sourcedata/eeg/ as non-BIDS originals.

#### Leap motion

For each run and hand, two source behavioral time series are provided under sub-XX/sourcedata/beh/: (i) a screen-stimulus stream (*_ball.tsv.gz) containing time, ball_x_pix, ball_color, move_direct, trial, and hit; and (ii) a hand-kinematics stream (*_leap.tsv.gz) with time, x_mm, y_mm, and (if available) z_mm. Sidecar JSON files document units and sampling (≈60 Hz for screen, 170 Hz for hand). Files in sourcedata/ are intentionally excluded from BIDS validation via.bidsignore but are provided to facilitate reprocessing and cross-modal alignment.

### Derived data

**Pre-movement EEG** *(stored under IACKD-BIDS/derivatives/premove/sub-XX/ihc/)*. Per run, files pre_eeg_L*.mat, pre_eeg_R*.mat contain −1.0 s EEG segments and associated metadata.

Each file includes:

- color_ball, color_code: Ball color for each trial and its categorical code (0 = red, 1 = yellow).
- move_direct, direct_code: Target movement direction for each trial and its categorical code (0 = left, 1 = right).
- label_code: Four-condition label (0–3): 0 = red-left, 1 = red-right, 2 = yellow-left, 3 = yellow-right.
- labels: A detailed lookup table linking each trial’s codes to the ball color, target direction, and the corresponding label_code.
- keep_mask_1s: Boolean mask indicating whether the extracted pre-movement segment meets the 1-s length requirement for validity (used as an intermediate variable in preprocessing).
- meta: Trial-level metadata structure including: resting-state segment length (ms), number and names of valid channels, and preprocessing parameters (down-sampling rate, band-pass range, removed channels, ICA threshold/criteria, re-reference mode, segment duration in ms, and the min/max allowable duration thresholds).
- segs_1s, times_1s: Fixed 1-s EEG windows (−1.0 to 0 s) and corresponding time vectors.
- segs_var, times_var: Variable-length pre-movement EEG and time axes covering the full 55 → 14 interval, aligned so 0 s = mark 14.
- trial_csv, trial_info: Trial index and trial-level metadata, including event/mark labels and their timestamps, inter-mark intervals, and a validity flag for each pre-movement segment.

**Aligned EEG-hand time series** *(stored under IACKD-BIDS/derivatives/aligned/sub-XX/ihc/)*. For each run, files eeg_leap_L*.mat and eeg_leap_R*.mat provide a MATLAB struct array OUT (one element per aligned trial) with the following key fields:

- eeg_seg_index: Index of the valid EEG segment used for alignment.
- im_trial: Corresponding Leap Motion trial index.
- t_ms (ms): Trial-specific time axis relative to EEG event 14, including a −200 ms baseline.
- EEG (µV; nChan × T; 100 Hz): Baseline-corrected EEG segment aligned to t_ms.
- x_mm, y_mm, z_mm (mm; 1 × T; 100 Hz): 3D hand position resampled to t_ms and smoothed with a zero-phase 4 Hz Butterworth filter.
- ball_x_pix (px; 1 × T; 100 Hz): Screen-space horizontal trajectory of the controlled ball.
- ball_color, move_direct: Trial-level labels indicating the color of the controlled ball and the target movement direction for the current trial.
- a, b: Affine parameters mapping EEG time (s) onto the Leap Motion global time axis.
- start_res_ms (ms): Residual onset offset for the current trial.
- cov (0–1): Temporal coverage fraction of the EEG segment within the corresponding Leap Motion trial.

All data are stored as MATLAB arrays or cell arrays with consistent naming and units, facilitating direct reuse in downstream EEG-kinematic analyses.

The OpenNeuro repository contains a BIDS-organized release of the raw EEG data (BrainVision files under sub-*/eeg/ with per-run *_events.tsv), the source behavioral time series under sub-*/sourcedata/beh/ (screen “ball” and hand “leap” streams with sidecar JSON), and two derivative collections (derivatives/premove and derivatives/aligned).

This folder contains the MATLAB scripts used for EEG and Leap Motion preprocessing and alignment. An accompanying README.md describes each script’s purpose, required inputs and outputs, and usage. The source code is also available at: https://github.com/Boketto1/IACKD.

#### Preprocessing scripts

This folder contains the MATLAB scripts used for EEG and Leap Motion preprocessing and alignment. An accompanying README.md describe each script’s purpose, required inputs and outputs, and usage. The source code is also available at: https://github.com/Boketto1/IACKD.

#### Units, and encoding

Aligned trial axes use t_ms relative to EEG event **14**; Leap timestamps are standardized to **seconds** during preprocessing. EEG amplitudes are in **microvolts (µV)**; hand positions in **millimeters (mm)**; screen coordinates in **pixels (px)**. Missing data outside the Leap coverage are encoded as NaN. Run naming follows L1-L4 (left) and R1-R4 (right) conventions across subjects.

## Technical Validation

### Readiness potentials (RPs)

**Fig. 4:**
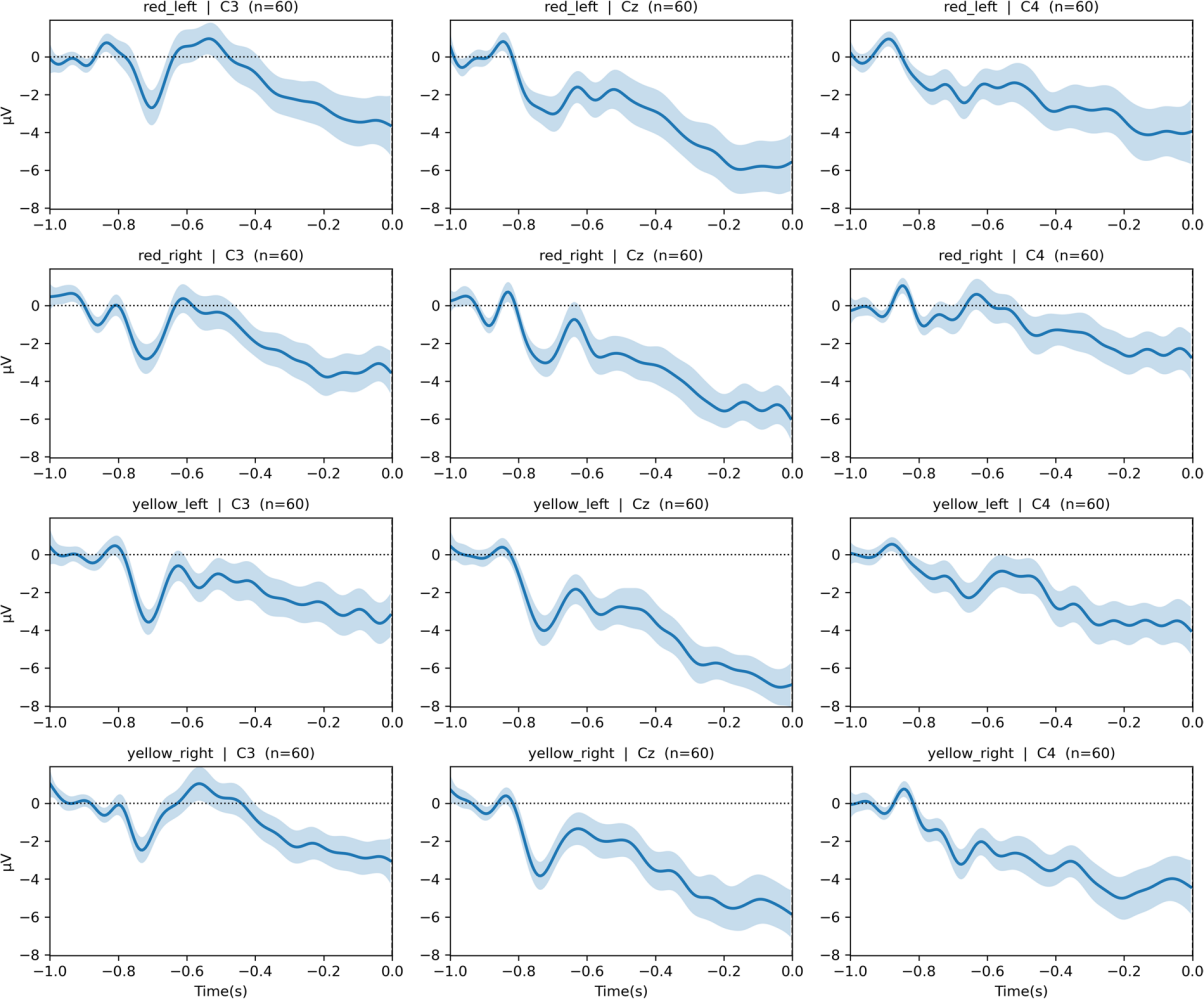
Readiness potentials during pre-movement at C3, C4, and Cz. Grand-average waveforms (mean ± 95% CI) over −1 to 0 s for four conditions (congruent-left/right, incongruent-left/right) sampled at 200 Hz, low-pass 10 Hz, baseline −1.0 to −0.8 s. All conditions show a slow-rising negative deflection starting around −0.8 s and peaking at movement onset, with the largest amplitude at Cz and similar temporal profiles at C3/C4, consistent with robust motor preparatory activity.

Readiness potentials (RPs) during the pre-movement period were analyzed to confirm the physiological validity of the EEG recordings. For each of the four task conditions (congruent-left, congruent-right, incongruent-left, and incongruent-right), averaged waveforms were obtained from electrodes C3, C4, and Cz within the −1 to 0 s window (200 Hz sampling rate). After preprocessing (0.1–45 Hz), the averaged RP waveforms were additionally low-pass filtered at 10 Hz for visualization purposes, in order to emphasize slow cortical potentials and suppress residual high-frequency noise. The baseline window was selected to precede the RP onset while reducing slow drifts, thereby improving comparability across trials and conditions. Figure 4 visualizes the pre-movement RPs for participant S03 during left-hand movement. At the group level, similar RP waveforms were consistently observed across participants in all four conditions, characterized by a gradual negative shift starting approximately 0.8 s before movement onset and peaking around the onset. Cz generally showed the largest deflection, consistent with contributions from midline motor areas. The RP exhibited an early bilateral distribution over C3 and C4, followed by a stronger negativity over the contralateral sensorimotor cortex close to movement onset, in line with classical motor preparation dynamics. Clear RPs were observed across all conditions, indicating that the pre-movement EEG segments reliably captured motor preparatory and predictive processes and that the event markers were temporally well aligned. No systematic RP amplitude or latency differences were observed between congruent and incongruent conditions, suggesting that RPs primarily reflect movement preparation rather than conflict-related mismatch or error processing. This analysis was implemented in Python in the script *rp4pre_move.py*.

### Cross-modal synchronization accuracy

**Fig. 5:**
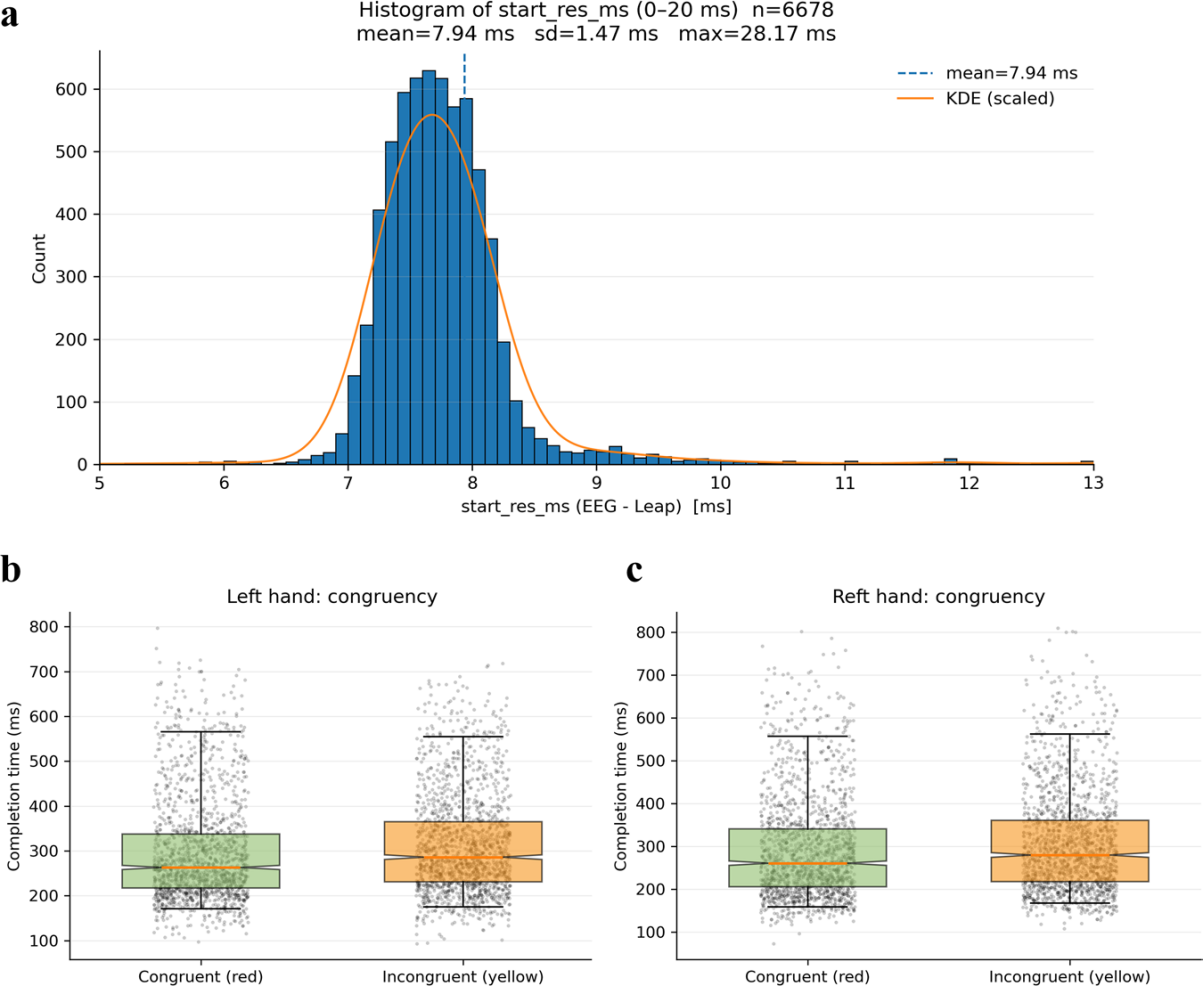
Cross-modal synchronization and completion-time characteristics. (**a**) Distribution of residual onset offsets (**start_res_ms**) between EEG activity onset and Leap Motion movement onset (trials with |offset| > 30 ms excluded). Mean ± s.e.m. reported in the panel (e.g., 7.94 ± 1.47 ms) demonstrates stable synchronization. (**b,c**) Completion times across participants for left-hand (**b**) and right-hand (**c**) movements under congruent (red) and incongruent (yellow) conditions.

To assess synchronization precision between EEG and motion-capture data, we quantified the temporal residual (start_res_ms) between corresponding temporal anchors in the EEG and Leap Motion streams after linear time calibration. In the EEG stream, trial timing was referenced to event marker 14, generated when the “Pre Move” cue disappeared, and to event marker 66, indicating trial completion/hit. In the Leap Motion stream, the corresponding anchors were defined as the first recorded frame of each trial and the first hit event when available. These paired anchors were used to calibrate the temporal correspondence between EEG time and Leap Motion global time. Figure 5a summarizes these results. Offsets exceeding ±30 ms were excluded. The residual distribution was narrowly centered near 7.94 ms, with a mean signed error below 1.47 ms and over 99.375% of trials within ±30 ms. This tight distribution demonstrates that the EEG and kinematic streams were precisely synchronized, ensuring temporal accuracy suitable for sensorimotor analyses. The synchronization and completion-time analyses were both implemented in the Python script *res_completion_time.py*.

### Completion-time characteristics

Movement completion time was defined as the duration from movement onset to trajectory termination in each trial (Fig. 5b,c). Across 15 participants, completion times ranged from approximately 190 to 560 ms, with mean durations of 303.84 ± 116.74 ms for left-hand and 299.98 ± 121.17 ms for right-hand movements, indicating strong inter-hand consistency.

To examine task congruency effects, trials were grouped by color cue: red for congruent (ball and hand moved in the same direction) and yellow for incongruent conditions. Averaged across subjects, congruent trials exhibited a mean completion time of 294.53 ± 119.52 ms, while incongruent trials were slightly slower (309.23 ± 117.03 ms), which may indicate a modest increase in processing demands under incongruent visuomotor mappings. These results confirm the temporal coherence and behavioral reliability of the recorded data.

### Neural and behavioral signal validation

To validate the neurophysiological integrity of the EEG data and its correspondence to movement behavior, we computed the event-related spectral perturbation (ERSP), inter-trial coherence (ITC), and mean hand trajectories under four movement conditions. EEG data were resampled to 100 Hz, band-pass filtered between 0.1 and 30 Hz, and analyzed using Morlet wavelets (2–30 Hz, 3–7 cycles). All trials were baseline-corrected within the −0.2 to 0 s window relative to movement onset. Although this interval is close to movement onset and may include preparatory activity, it was chosen to ensure consistent alignment to onset across trials and to reduce low-frequency drifts. Therefore, ERSP values should be interpreted as relative modulations rather than changes from a resting baseline. For ERSP analysis, time-frequency power at each time-frequency point was normalized to the mean baseline power at the corresponding frequency using the −0.2 to 0 s interval relative to movement onset as baseline. Specifically, baseline correction was applied in log-ratio form:

$${ERSP}(t,f)=\log \left(\frac{P(t,f)}{{P}_{{base}}(f)}\right)$$

where $P(t,{f})$ denotes the time-frequency power and ${P}_{{base}}(f)$ denotes the mean baseline power at frequency *f* within the baseline interval. Although this interval is close to movement onset and may include preparatory activity, it was chosen to ensure consistent alignment to onset across trials and to reduce low-frequency drifts. Therefore, ERSP values should be interpreted as relative modulations rather than changes from a resting baseline. ITC was computed without baseline correction. Analyses focused on the C4 channel, which is most sensitive to contralateral motor activity.

**Fig. 6:**
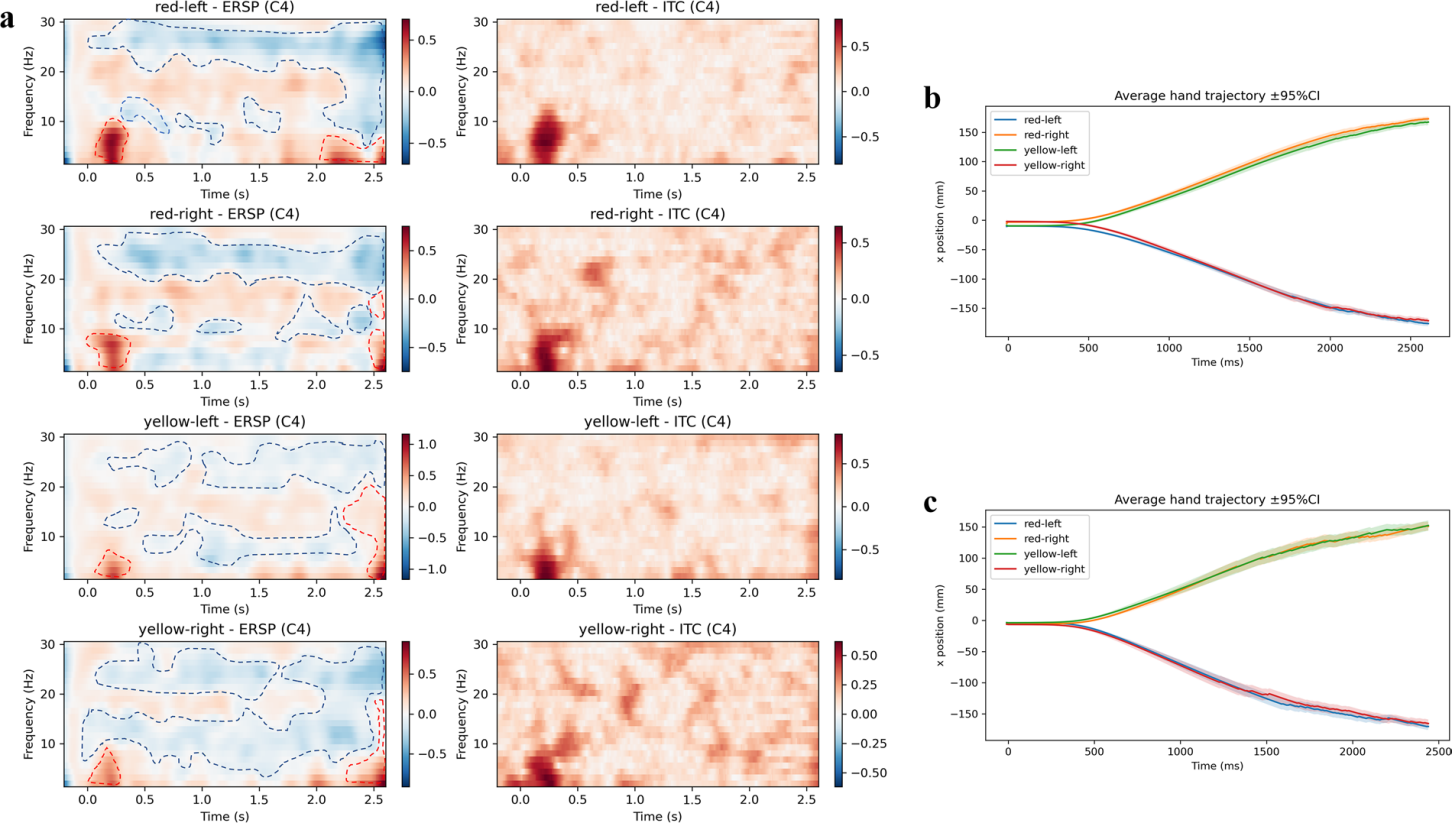
Spectral-phase metrics and hand trajectories. (**a**) ERSP and ITC at C4 for participant S03 (left-hand execution). The μ/β-band desynchronization (ERD) is highlighted by blue dashed contours, and post-movement rebound-related activity is highlighted by red dashed contours in all ERSP plots, together with transient phase-locking around movement onset. (**b,c**) Mean x-axis trajectories (±95% CI) for left- and right-hand movements, respectively, illustrating directional separation and comparable kinematic smoothness across congruency conditions.

Figure 6a shows ERSP and ITC maps for participant S03 during left-hand execution across the four conditions: robust μ/β (α: 8–13 Hz; β: 13–30 Hz) desynchronization preceded movement, followed by post-movement rebound-related activity extending beyond the canonical beta range; ITC exhibited transient phase-locking near onset. Figure 6b,c depict averaged hand trajectories (±95% CI) along the x-axis for left- and right-hand movements, respectively. Trajectories were smooth and monotonic, with clear separation between movement directions; congruent and incongruent trials traced distinct spatial paths while maintaining comparable kinematic smoothness, supporting accurate temporal alignment between EEG and motion capture. This analysis was implemented in Python (*ersp_itc_trajectory.py*).

Together, the ERSP, ITC, and trajectory analyses verify the temporal precision, physiological interpretability, and cross-modal consistency of the dataset, providing a robust foundation for future studies on sensorimotor decoding and brain-computer interaction modeling.

## Data Availability

The dataset described in this study is publicly available on OpenNeuro under accession number **ds006840** and can be accessed via its 10.18112/openneuro.ds006840.v1.0.0.

## Code availability

The source code used for all technical validations conducted in this experiment has been uploaded and is publicly available on GitHub (https://github.com/Boketto1/IACKD).
